# Mitral valve repair in patient with absent right superior vena cava in visceroatrial situs solitus

**DOI:** 10.1186/1749-8090-8-9

**Published:** 2013-01-15

**Authors:** Toshiro Kobayashi, Takeshi Yagi, Yoshikazu Okazaki, Mitsutaka Jinbo, Satoshi Saito, Tsuyoshi Takahashi, Hidenori Gohra

**Affiliations:** 1 Departments of Surgery, Saiseikai Yamaguchi General Hospital, 2-11 Midori-Cho, Yamaguchi, Yamaguchi, 753-0078, Japan

**Keywords:** Absent right superior vena cava, Persistent left superior vena cava, Visceroatrial situs solitus, Mitral valve plasty

## Abstract

We report on a 74-year-old woman with an absence of right superior vena cava in visceroatrial situs solitus who underwent mitral valve plasty for severe mitral regurgitation. Preoperative three-dimensional computed tomography revealed an absent right and persistent left superior vena cava that drained into the right atrium by way of the coronary sinus. Perioperaively, placement of pulmonary artery catheter, site of venous cannulation, and management of associated rhythm abnormalities were great concern. Obtaining the information about this central venous malformation preoperatively, we performed mitral valve plasty without any difficulties related to this anomaly.

## Background

Persistent left superior vena cava (PLSVC) with absent right superior vena cava (RSVC) in visceroatrial situs solitus is an extremely rare congenital anomaly [1,2]. This anomaly can exist alone, and is difficult to diagnose because the hemodynamics of patients with this condition are normal and there may be lack of clinical symptoms. It is often discovered accidentally while undergoing through examination for heart disease, during pacemaker implantation, or central venous catheter insertion. Before the surgical intervention, this anomaly should be diagnosed correctly to avoid any complications. We report one patient with this anomaly who underwent mitral valve plasty for severe mitral regurgitation.

## Case presentation

**Figure 1:**
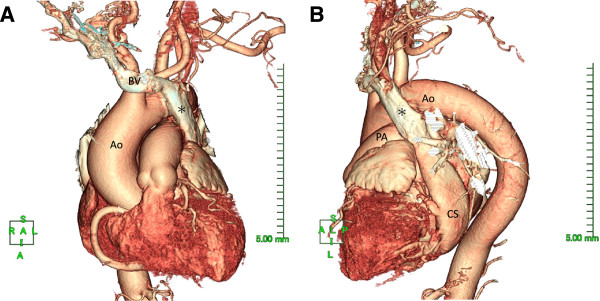
**Contrast enhanced CT Thorax scan with the 3D reconstruction of the heart; 1A: anterior view; 1B: left lateral view.** The left sided superior vena cava (PLSVC: *) is being formed by the bridging vein (BV), which drains the jugular and subclavian vein. The left brachiocephalic vein is not present since contrast medium was injected via a right cubital vein. PLSVC descended at the left side of the mediastinum leftward of the pulmonary artery and left atrium before draining into the right atrium via a dilated coronary sinus. Ao: Aorta, PA: Pulmonary artery, CS: Coronary sinus.

A 74-year-old female was admitted to other hospital with facial and leg edema and presented an exertional dyspnea. Transthorasic echocardiogram (TTE) at that time showed severe mitral regurgitation. She was treated medically and had regular follow-up with serial echocardigrams. Over the period her exercise tolerance deteriorated. Echocardiogram showed gradual increasing left ventricular end-diastolic dimension and decreasing left ventricular ejection fraction. She was referred for mitral valve surgery on basis of severe mitral regurgitation. Preoperative transesophageal echocardiogram (TEE) showed severe mitral regurgitation secondary to prolapse of posterior mitral valve leaflet. Cardiac catheterization revealed normal coronary arteries. A contrast enhanced multi-detector computed tomography (MDCT) showed a bridging vein draining the right jugular and right subclavian veins; it joined the left brachiocephalic vein and formed the PLSVC, which descended at the left side of the mediastinum leftward of the pulmonary artery and left atrium before draining into the right atrium via a dilated coronary sinus. The RSVC was absent and the PLSVC carried all venous blood from the head, neck and upper extremities (Figure 1). There was no other pathological finding. The visceral organs were normally positioned.

Our perioperative management plans were as follows; (1) placement of a pulmonary artery catheter through a right femoral vein under fluoroscopy; (2) insertion of a venous drainage cannula through the PLSVC itself and inferior vena cava; and (3) placement of temporary pacing wires to the right ventricle to manage the associated rhythm abnormalities.

**Figure 2:**
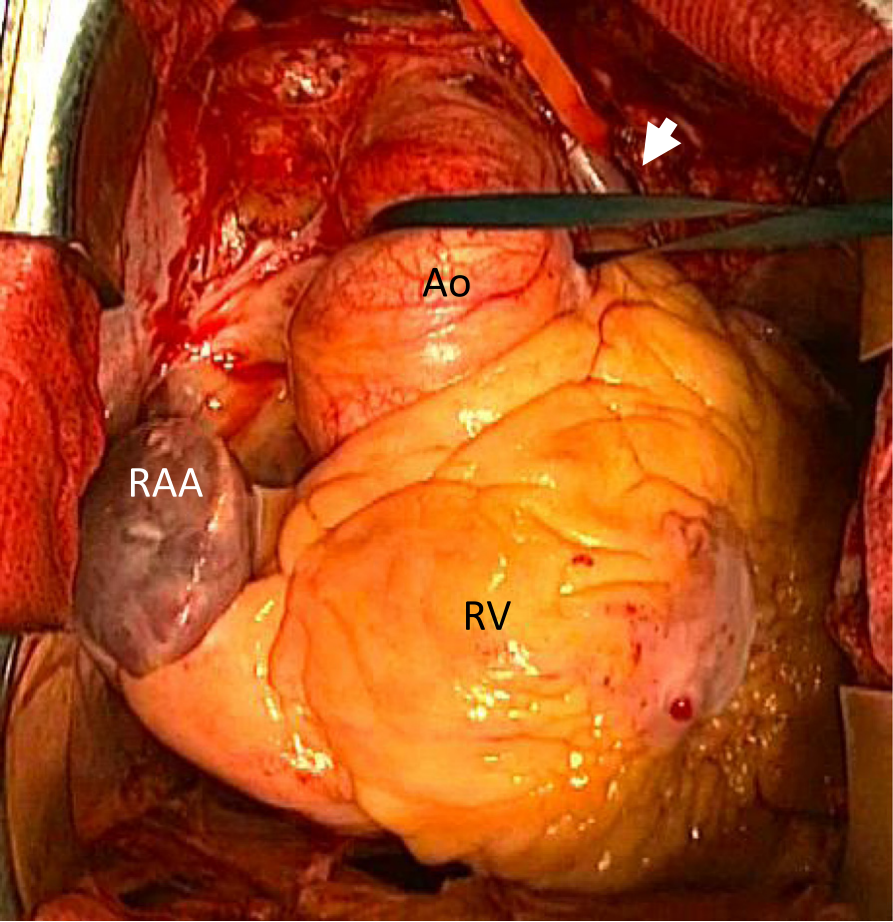
**Operative finding showed a complete absence of the right superior vena cava.** Ao: Ascending aorta, RAA: Right atrial appendage, RV: Right ventricle. Arrowhead: PLSVC.

Median sternotomy and pericardiotomy was made. The right-sided appendage displayed the morphology of right atrium and left-sided one of left, and inferior vena cava entered the right-sided atrium. The bridging vein ran toward left and connected to the PLSVC, which flowed to the coronary sinus in the right atrium. The RSVC was completely absent (Figure 2). Cardiopulmonary bypass was established with ascending aortic cannulation and venous drainage directly from PLSVC and IVC with L-shaped cannulas. Antegrade cardioplegia was used for myocardial protection. After opening the right side of the left atrium, a close examination of the mitral valve showed extensive posterior leaflet prolapse in P2 segment due to rupture of primary chordae. Mitral valve repair was performed with quadrangular resection and annular plication following mitral ring annuloplasty. Although preoperative echocardiography of this patient showed a sinus rhythm, temporary pacing wires placed to the right ventricle. The postoperative course for the patient was uneventful and echocardiography showed only trivial mitral regurgitation.

## Discussion

Persistent left superior vena cava with absent RSVC in visceroatrial situs solitus is extremely rare, occurring in 0.07 to 0.15% in patient with transvenous pacemaker or defibrillator implantations, 0.09 to 0.13% in postmortem cases of congenital heart disease [1-3]. Although absence of RSVC in patients with visceroatrial situs solitus is by itself of no hemodynamic significance, its diagnosis before surgery or other invasive procedure is important to avoid various management difficulties, which include the following. (1) Implantation of a transvenous pacemaker or defibrillator. (2) Placement of pulmonary artery catheter for intraoperative or intensive care unit monitoring. (3) Systemic venous cannulation for cardiopulmonary bypass or extracorporeal circulation, (4) Cavopulmonary anastomosis, and (5) Orthtopic heart transplantation [1,2]. The diagnosis can be confirmed by TTE, TEE, venous angiography, computed tomography or magnetic resonance imaging. Preoperative MDCT is useful for detection and directly visualizing for whole image of these venous anomalies [4,5]. In this particular case, our perioperative strategy regarding the placement of pulmonary artery catheter and the venous cannulation site in preparation for cardiopulmonary bypass seemed to be reasonable. A venous cannula can be inserted into PLSVC in either way: a cannula can be inserted from the coronary sinus, or an L-shaped cannula can be directly inserted into the PLSVC. Direct insertion of cannula into coronary sinus would be dangerous from the standpoint of coronary sinus injury [6]. In this patient, PLSVC was well exposed so taping and cannulation can be carried out without any difficulty, it is no need for cannulation to PLSVC through coronary sinus.

A higher incidence of arrhythmia and conduction system abnormalities has been described in patients with PLSVC. A dilated coronary sinus stretches the atrioventricular nodal tissue, or the early conduction tissue has close proximity to the cardinal venous tissue and this leads to sinus node dysfunction [7,8]. This case did not demonstrate abnormalities of cardiac conduction system preoperatively, although all these rhythm abnormalities are not necessarily related to those venous anomalies itself, careful management in certain types of arrhythmias is need perioperatively.

## Conclusion

We report our case of treating a patient with the absent RSVC in visceroatrial situs solitus who underwent mitral valve plasty. Preoperative MDCT is useful non-invasive imaging modality for detection of central venous anomaly. Perioperative strategy including placement of pulmonary artery catheter, selection of venous cannulation site in preparation for cardiopulmonary bypass and control of associated supraventricular rhythm abnormalities leads successful management and good clinical result.

## Consent

Written informed consent was obtained from the patient for publication of this Case report and any accompanying images. A copy of the written consent is available for review by the Editor-in-Chief of this journal.

## Competing interest

All the authors declare that they have not competing interest.

## Authors’ contributions

In the following we specify the individual contributions of authors to the manuscript. The mitral valve plasty and all surgical procedures were performed by TK, YO and HG. TY, MJ, SS and TT managed the perioperative period of the patient. TK prepared the manuscript and HG made final approval. All authors read and approved the final manuscript.
